# Dissociating Memory Networks in Early Alzheimer’s Disease and Frontotemporal Lobar Degeneration - A Combined Study of Hypometabolism and Atrophy

**DOI:** 10.1371/journal.pone.0055251

**Published:** 2013-02-14

**Authors:** Stefan Frisch, Juergen Dukart, Barbara Vogt, Annette Horstmann, Georg Becker, Arno Villringer, Henryk Barthel, Osama Sabri, Karsten Müller, Matthias L. Schroeter

**Affiliations:** 1 Department of Neurology, J. W. Goethe University, Frankfurt/Main, Germany; 2 Max-Planck-Institute for Human Cognitive and Brain Sciences, Leipzig, Germany; 3 LREN, Département des Neurosciences Cliniques, CHUV, Lausanne, Switzerland; 4 Department of Nuclear Medicine, University of Leipzig, Leipzig, Germany; 5 Day Clinic of Cognitive Neurology, University of Leipzig, Leipzig, Germany; 6 LIFE - Leipzig Research Center for Civilization Diseases, University of Leipzig, Germany; 7 German FTLD Consortium, Germany; Banner Alzheimer's Institute, United States of America

## Abstract

**Introduction:**

We aimed at dissociating the neural correlates of memory disorders in Alzheimer’s disease (AD) and frontotemporal lobar degeneration (FTLD).

**Methods:**

We included patients with AD (n = 19, 11 female, mean age 61 years) and FTLD (n = 11, 5 female, mean age 61 years) in early stages of their diseases. Memory performance was assessed by means of verbal and visual memory subtests from the Wechsler Memory Scale (WMS-R), including forgetting rates. Brain glucose utilization was measured by [18F]fluorodeoxyglucose positron emission tomography (FDG-PET) and brain atrophy by voxel-based morphometry (VBM) of T1-weighted magnetic resonance imaging (MRI) scans. Using a whole brain approach, correlations between test performance and imaging data were computed separately in each dementia group, including a group of control subjects (n = 13, 6 female, mean age 54 years) in both analyses. The three groups did not differ with respect to education and gender.

**Results:**

Patients in both dementia groups generally performed worse than controls, but AD and FTLD patients did not differ from each other in any of the test parameters. However, memory performance was associated with different brain regions in the patient groups, with respect to both hypometabolism and atrophy: Whereas in AD patients test performance was mainly correlated with changes in the parieto-mesial cortex, performance in FTLD patients was correlated with changes in frontal cortical as well as subcortical regions. There were practically no overlapping regions associated with memory disorders in AD and FTLD as revealed by a conjunction analysis.

**Conclusion:**

Memory test performance may not distinguish between both dementia syndromes. In clinical practice, this may lead to misdiagnosis of FTLD patients with poor memory performance. Nevertheless, memory problems are associated with almost completely different neural correlates in both dementia syndromes. Obviously, memory functions are carried out by distributed networks which break down in brain degeneration.

## Introduction

Memory problems are the most frequent complaint by patients presenting for clinical assessment when dementia is suspected. Although disorders of memory have been defined as a necessary criterion in the diagnosis of dementing illness (ICD-10; DSM-III), these definitions have largely been based on the most frequent dementia syndrome - Alzheimer's disease (AD). Memory impairments have been claimed to play a subordinate role in other forms of neurodegenerative disorders, such as frontotemporal lobar degeneration (FTLD). Here, memory has been assumed to remain largely intact at least in early stages of the disease [1], [2] in contrast to other cognitive domains such as behavioral abnormalities, executive and language deficits, depending on the subtype [3]–[5]. The finding that AD patients face more problems in anterograde episodic memory than FTLD patients has been confirmed in several studies [6]–[13], although there is accumulating evidence that memory can be remarkably impaired even in early stages of FTLD [14]–[17]. One explanation of this latter finding is that temporo-mesial/hippocampal atrophy is also found in FTLD, though more asymmetric (left>right) than with AD [18]–[22]. Accordingly, it has been claimed that FTLD cannot be clearly distinguished from AD on the basis of memory test performance [23]–[27], at least not on the individual level relevant for diagnostic classification [28]. Additionally, methodological issues such as differences in lesion patterns, verification of diagnosis, different test measures etc. further complicate the comparison of studies [29], [30]. With respect to the nature of their memory problems, FTLD patients were supposed to show more ‘frontal’ deficits such as problems in organizing material that is to be learned [8], [11], [29], [31] or in retrieving information rather than storing it [11], [12], [24], [29]. By contrast, AD patients have been claimed to exhibit stronger consolidation problems as they seem to lose more information over time [11], [24]. Other studies have shown stronger visual memory problems in AD patients probably due to additional visuo-spatial problems which cannot be compensated for by verbal memory strategies [14], [18].

Furthermore, a number of studies have related memory performance in dementia patients directly to the lesion patterns as revealed by imaging techniques -such as Magnetic Resonance Imaging (MRI) and [18F] FluoroDeoxyGlucose Positron Emission Tomography (FDG-PET)- in order to explain variability across studies. AD leads to atrophy and/or hypometabolism in temporo-mesial [32]–[35] as well as parieto-mesial areas [35]–[39] as early signs of the disease. Accordingly, several studies (e.g. [40], [41]) have shown that in patients with amnestic mild cognitive impairment (aMCI), a prestage of AD, deficits in word list encoding and retrieval correlate with hippocampal atrophy as well as with hippocampal and parieto-medial hypometabolism (precuneus, posterior cingulate). Correlations of memory deficits and hippocampal atrophy in AD have been confirmed in several studies [42]–[44]. Using Single-photon Emission Computed Tomography (SPECT), Caroli and colleagues [45] found that hypoperfusion in the retrosplenial cortex and precuneus was associated with memory impairment, but that only hippocampal hypoperfusion predicted a conversion of MCI to AD. Desgranges and colleagues [46], [47] found correlations between lower performance in verbal memory (word list, story recall) and PET hypometabolism in the temporo-medial cortex, thalamus, cingulate and parieto-medial areas, but also in lateral temporal, parietal and frontal cortices in AD patients (see also ref [39]). Furthermore, it has been shown that hippocampal hypometabolism affects the recall of (at least recent) episodes from autobiographical memory in AD [48].

By contrast, there are only very few studies investigating the relationship between hypometabolism and/or atrophy and memory functions in patients with FTLD. In semantic dementia, hypometabolism and atrophy are found especially in left lateral temporal areas [20], [22], [49], [50], but also in the hippocampus [50], [51] suggesting that memory is more severely affected than previously assumed (see above). As shown in a further study [52], frontal-variant FTLD patients (‘frontotemporal dementia’ according to [1]) had difficulties in retrieving autobiographical memories compared to control subjects (see also [17]). These difficulties were related to orbitofrontal as well as temporal hypometabolism.

To our knowledge, there is only one recent study [53] in which memory performance in AD and FTLD has been linked to brain atrophy. Using atrophy ratings, the authors found that neural correlates differed (temporal and frontal areas in AD vs. frontal in FTLD), although memory performance was alike between both dementia groups. However, no previous study has systematically explored the neural correlates of memory in early AD and FTLD patients with respect to both regional hypometabolism as measured with [18F]fluorodeoxyglucose positron emission tomography (FDG-PET) as well as brain atrophy as measured with voxel-based morphometry (VBM) of magnetic resonance imaging (MRI) in a whole brain approach. This was the aim of the present study.

Based on the studies outlined above, we drew the following hypotheses: General lesion patterns as revealed by hypometabolism and atrophy should differ between the two syndromes, with a parietotemporal distribution in AD and frontotemporal in FTLD [22]. With respect to memory performance, previous studies did not allow to clearly predict group differences in encoding or retrieving information, but probably with respect to forgetting rates as some studies found a greater loss of information over time in AD [11], [24]. Nevertheless, we expected the neural correlates of memory to differ between the groups according to the differences in lesion patterns [53], which include memory relevant areas in both diseases.

## Materials and Methods

### Subjects

Subjects were recruited from the Day Clinic of Cognitive Neurology at the University of Leipzig. Our study included 19 patients with an early stage of probable AD and 11 patients with an early stage of FTLD (frontotemporal dementia (FTD) n = 4, semantic dementia (SD) n = 5, mixed type n = 2). Probable AD was diagnosed according to NINCDS-ADRDA (National Institute of Neurological and Communicative Disorders and Stroke- Alzheimer’s Disease and Related Disorders Association) criteria [54]. All AD subjects also fulfilled the revised NINCDS-ADRDA criteria [55]. Diagnosis of FTLD was based on the criteria of ref [1]. Symptom severity was measured with the Clinical Dementia Rating Scale (CDR) [56].

We included 13 controls who presented at the clinic because of subjective cognitive complaints, which were not objectively confirmed by a comprehensive neuropsychological and clinical evaluation. Thus, these subjects had noticed normal, age-related decreases in cognitive performance which concerned them. Therefore, they presented at the clinic and asked for a screening for dementia. Their CDR scores ranged between 0 and 0.5.

FDG-PET and MRI as well as the neuropsychological assessment were conducted for diagnostic reasons within the clinical assessment. Subjects were excluded if structural imaging revealed lesions due to stroke, traumatic brain injury, brain tumor or inflammatory diseases. Diagnoses were made prior to computing all subsequent analyses, i. e. the group differences as well as the correlations between test measures and imaging parameters. As can be seen from Table 1, CDR scores indicated early dementia stage for both AD and FTLD. Furthermore, all three groups differed significantly with respect to age (F = 5.3; p<.01) and CDR (F = 16.2/p<.001), but neither with respect to years of education (F<1) nor gender (χ^2^ = .62; p = .73). We found differences between each of the dementia groups versus the controls with respect to age (AD vs. Controls: t(30) = 2.9; p = .016; FTLD vs. Controls: t(22) = 2.9; p = .028) and CDR (AD vs. Controls: t(30) = 5.2; p<.001, FTLD vs. Controls: t(22) = 4.7; p<.001). Therefore, age was included as a covariate to control for a possible confounding impact of age on the outcome measures. AD and FTLD patients did not differ from one another with respect to age (t(28) = −.5; p>.99) and CDR (t(28) = −.2; p>.99).

**Table 1:** Subject characteristics and test results for each group.

	Controls	AD	FTLD	GLM (F/p)
n	13	19	11	
Gender (m/f)	7/6	8/11	6/5	?^2^ = .62/p = .73
Age (ys)	53.92 (6.00)	60.89 (6.94)	61.27 (6.53)	5.3/.009
Education (ys)	12.31 (3.14)	10.95 (3.1)	11.64 (3.88)	.7/.526
CDR (score)	.23 (.26)	.71 (.25)	.73 (.26)	16.2/<.001
WMS-LM1	28.62 (4.82)	11.47 (6.75)	13.10 (6.06)	24.4/<.001
WMS-LM2	23.54 (8.41)	6.77 (6.07)	11.70 (6.99)	14.9/<.001
WMS-LMFR (%)	19.11 (21.57)	26.36 (90.17^1^)	18.34 (32.16)	.1/.924
WMS-VR1	38.33 (2.10)	23.95 (7.31)	29.7 (9.41)	12.7/<.001
WMS-VR2	34.42 (6.34)	13.42 (10.75)	21.5 (13.52)	9.6/<.001
WMS-VRFR (%)	10.07 (17.14)	49.28 (34.14)	35.89 (36.89)	3.5/.042

Means are given with standard deviations in parentheses. AD: Alzheimer’s disease, FTLD: frontotemporal lobar degeneration, CDR: Clinical Dementia Rating Scale, GLM: General Linear Model, WMS: Wechsler Memory Scale, LM: Logical Memory 1: immediate, 2: delayed, FR: forgetting rate), VR: Visual Reproduction 1: immediate, 2: delayed, FR: forgetting rate). For further analyses see text.

1 The high standard deviation in this test measure was due to the fact that one AD patient recalled more information in the delayed compared to the immediate recall, although on an extremely low level of performance (one item in the immediate and four items in the delayed recall, thus resulting in a forgetting rate of −300).

### Ethics Statement

Written informed consent was obtained from subjects to analyse their diagnostic data retrospectively. In cases in which the ability to consent was already compromised as the disease had progressed, consent was obtained by caregivers (family members). The research protocol was approved by the ethics committee of the University of Leipzig, and was in accordance with the latest version of the Declaration of Helsinki.

### Neuropsychological Assessment

Memory performance in the present study was assessed with two subtests from the German version of the revised Wechsler Memory Scale (WMS-R) [57]: In the subtest *Logical Memory* (LM), two brief stories are read to the subject by the examiner. The participant is asked to recall as many details as possible of each story, both immediately following each presentation (Logical Memory I, maximum score 50) and after a delay of approximately 30 minutes (Logical Memory II, maximum score 50). In the *Visual Reproduction* (VR) subtest, subjects are asked to recall four briefly presented abstract figures by drawing them from memory. As in the LM subtest, the VR figures are to be recalled both immediately after each presentation (Visual Reproduction I, maximum score 41) and after a 30 min delay (Visual Reproduction II, maximum score 41). The WMS-R is widely used for the assessment of memory deficits and has been investigated extensively [58]. In meta-analyses, several WMS subtests have proven to dissociate AD patients from normal controls, especially with respect to delayed recall measures [59]. Furthermore, the *Visual Reproduction* subtest might be useful to distinguish AD from FTLD patients [14]. In addition to the original immediate and delayed recall measures of each subtest, we computed forgetting rates (FR) as this measure has been proposed to be of value in differentiating between AD and FTLD patients [11], [24]. Forgetting rates (FR) were computed as individually normalized values according to the following formula for LM and VR, respectively: FR = (immediate recall–delayed recall)/immediate recall*100. Some WMS-R data were unavailable for some of the subjects: LM was available for 17 AD patients, 10 FTLD patients and 13 control subjects and VR for all 19 AD patients, 10 FTLD patients and 12 control subjects. Three subjects (1 AD, 1 FTLD and 1 control subject) underwent the LM but not the VR subtest. For four subjects (3 AD and 1 FTLD), VR was available but not the LM subtest. Differences between the groups for each test (including FRs) were analyzed using general linear models (GLMs) with a factor Group (Control vs. AD vs. FTLD) and Age as a covariate. As age was not a significant predictor for any of the tests, the post-hoc analyses were computed only for the factor Group by using t-tests (p<.05) with a Bonferroni adjustment (in case of significance).

### Imaging Data Analysis

#### MRI data acquisition

For each subject, a high-resolution T1-weighted MRI scan was obtained, consisting of 128 sagittal slices adjusted to the AC (anterior commissure) - PC (posterior commissure) line with a slice thickness of 1.5 mm and a pixel size of 1×1 mm^2^. MRIs were performed on two different 3T scanners (MedSpec 30/100, Bruker Biospin, Ettlingen Germany and Magnetom Trio, Siemens, Erlangen, Germany) using two different T1-weighted sequences (MDEFT or MP-RAGE with TR = 1300 ms, TI = 650 ms, TE = 3.93 ms or TE = 10 ms; FOV 25×25 cm^2^; matrix  = 256×256 voxels). On the MedSpec scanner, only the MDEFT sequence and on the Magnetom Trio scanner, either MDEFT or MP-RAGE sequences were used. The distribution of scanner types and sequences used to obtain the MRI data was random across subjects and did not differ significantly in its distribution, neither between the groups nor between the scanner types nor between the sequences.

#### PET data acquisition

FDG-PET imaging was done either a few weeks before or after the MRI scan. PET data were acquired on a Siemens ECAT EXACT HR+ scanner (CTI/Siemens, Knoxville, TN, USA) under a standard resting condition in a 2-dimensional (2D) mode. Sixty-three slices were simultaneously collected with an axial resolution of 5 mm full width at half maximum (FWHM) and an in-plane resolution of 4.6 mm. After correction for attenuation, scatter, decay and scanner-specific dead-time, images were reconstructed by filtered back-projection using a Hann-filter of 4.9 mm FWHM. The 63 transaxial slices obtained had a matrix of 128×128 voxels with an edge length of 2.45 mm.

#### Preprocessing and imaging data analysis

Preprocessing was performed using Statistical Parametric Mapping (SPM 5, Wellcome Trust Centre for Neuroimaging) run with Matlab 7.7 (The MathWorks Inc., Natick, MA) applying the procedure described in detail in ref [60]: Co-registration and interpolation of MRI and FDG-PET images to a resolution of 1×1×1 mm^3^, partial volume effect correction of FDG-PET images using the modified Müller-Gärtner method [61], bias correction of MRI data, spatial normalization to a study specific template using the DARTEL approach (Diffeomorphic Anatomical Registration Through Exponentiated Lie algebra, cf. [62]), modulation of MRI data, masking of non gray matter voxels and smoothing using a 12 mm FWHM kernel. Intensity values of FDG-PET scans were normalized to the cerebellar values [63].

Whole brain correlation analyses were conducted between the obtained gray matter volume or glucose metabolism maps and associated scores of the WMS-R. As age differed significantly between the groups, it was included as a covariate in all correlation analyses. A significance threshold of p<.001 (uncorrected) at voxel-level and a cluster extent threshold of 2000 voxels were used in all analyses. The cluster threshold was used to exclude smaller clusters from the analysis and thereby decrease the risk of false positive errors. The high extent threshold results from the interpolation of the imaging data to a 1×1×1 mm^3^ resolution and corresponds to about 70 voxels in the original FDG-PET image which is in the range of commonly applied extent thresholds for this type of data. Correlation analyses in FDG-PET and MRI were performed for all six test scores (VR1, VR2, LM1, LM2, VRFR, LMFR) for either AD patients and controls or FTLD patients and controls, respectively. Additionally, to enable an evaluation of both glucose utilization and brain volume in dementia patients, group comparisons with respect to FDG-PET and MRI were computed over all 43 subjects to investigate differences between each group of dementia patients and control subjects, respectively. For the group comparisons, age, gender and total intracranial volume (only for MRI) were included as covariates. Additionally, we performed conjunction analyses to investigate similarities between observed imaging patterns correlating with single WMS-R measurements in the groups including either AD or FTLD patients.

## Results

### Neuropsychological Data

Table 1 depicts the test results for each patient group and the controls. As the ANOVA results show, the three groups differed significantly with respect to performance in LM1, LM2, VR1, VR2 and VRFR, but not LMFR. There were significant group differences between each of the dementia groups versus controls for LM1 (AD vs. Controls: t(28) = −7.8/p<.001, FTLD vs. Controls: t(21) = −6.8; p<.001), LM2 (AD vs. Controls: t(28) = −6.4; p<.001, FTLD vs. Controls: t(21) = −3.6; p = .001), VR1 (AD vs. Controls: t(29) = −6.6; p<.001, FTLD vs. Controls: t(20) = −3.1; p = .017), and VR2 (AD vs. Controls: t(29) = −6.1; p<.001, FTLD vs. Controls: t(20) = −3.0; p = .019). However, AD and FTLD patients did not differ from one another with respect to LM1 (t(25) = −.6; p>.99), LM2 (t(25) = −1.9; p = .27), VR1 (t(27) = −1.8; p = .13) and VR2 (t(26) = −1.6; p = .24). The main effect for VRFR was due to a difference between AD patients and control subjects (t(29) = 3.7; p = .004), but neither between FTLD patients and control subjects (t(20) = 2.2; p = .18) nor between AD and FTLD patients (t(26) = .8; p>.99). In sum, AD and FTLD patients performed worse than controls, but both dementia groups did not differ from one another. However, VRFR was significantly higher in AD, but not in FTLD, compared to control subjects.

### Imaging Results for Group Comparisons

**Figure 1:**
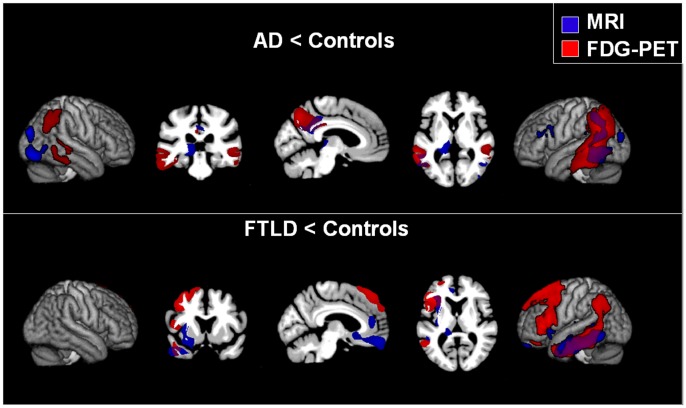
Group differences. Differences between AD patients and controls (upper part) as well as FTLD patients versus controls (lower part) in brain glucose utilization (FDG-PET, red) and atrophy (MRI, blue). Areas of overlap are shown in violet. Only clusters are shown that exceeded a significance threshold of p<0.001 uncorrected at voxel level and a cluster threshold of 2000 voxels. Left is left.

Figure 1 displays hypometabolism (FDG-PET) and atrophy (MRI) in AD and FTLD patients versus controls, respectively. Statistical results can be found in Table 2. As can be seen from the figure, AD patients showed a specific pattern of hypometabolism (FDG-PET) especially in the parieto-mesial cortex (precuneus, posterior cingulate), whereas hypometabolism in the FTLD group was mainly found in the left fronto-median and fronto-lateral cortex and in the insula. Hypometabolism in lateral temporal and inferior parietal regions was seen in both groups in comparison to controls. It was restricted to the left hemisphere in the FTLD group, but occurred bilaterally in the AD patients.

**Table 2:** Differences in hypometabolism and atrophy between groups.

	Regions	Cluster size	x	y	z	T-value
***FDG-PET***						
**AD<Con**	bilateral inferior precuneus, bilateral dorsal posterior cingulate gyrus, leftinferior, middle and superior temporal gyrus, left inferior parietal lobule	63163	−39	−54	39	6.8
	right inferior, middle and superior temporal gyrus, right inferior parietal lobule	16005	40	−55	22	6.2
**FTLD<Con**	left inferior, middle and superior frontal gyrus, left dorsal frontomediancortex, left superior anterior insula	34412	−39	28	8	4.94
	left inferior, middle and superior temporal gyrus, left inferior parietal lobule	21621	−55	−33	−17	4.75
***MRI***						
**AD<Con**	right middle occipital gyrus	3495	30	−80	19	6.28
	right inferior and middle occipital gyrus, right posterior inferior andmiddle temporal gyrus	4535	39	−75	−8	5.76
	bilateral dorsal posterior cingulate cortex, bilateral inferior precuneus,bilateral retrosplenial cortex	8822	1	−45	29	5.61
	left posterior superior, middle and inferior temporal gyrus, left inferiorparietal lobule/angular and supramarginal gyrus	17798	−44	−45	8	4.86
	left posterior hippocampus, left thalamus	4376	−18	−35	−1	4.83
	left middle occipital gyrus	2347	−29	−81	14	4.34
	left inferior frontal gyrus, left inferior frontal sulcus	2994	−45	17	21	4.3
**FTLD<Con**	left parahippocampal gyrus, left anterior hippocampus, left temporal pole, leftsubcallosal area, left anterior and posterior insula, left anterior cingulate cortex,left caudate head, left anterior putamen, left ventral striatum, left basalforebrain, left middle and inferior temporal gyri, bilateral rectal gyrus,bilateral inferior frontomedian cortex	35995	−52	−14	−18	5.91
	left posterior thalamus	2033	−17	−35	2	3.99

Statistical results for the comparisons between each dementia group and controls with respect to hypometabolism (FDG-PET) and atrophy (MRI). Cluster size is reported in voxels, voxel size was 1×1×1 mm.

Regional atrophy (MRI) was found in similar regions as hypometabolism and additionally in subcortical structures. In particular, gray matter (GM) loss in the (left) hippocampus and in the thalamus was found in both groups. By contrast, only FTLD patients exhibited atrophy in the insula, the basal ganglia and the basal forebrain relative to control subjects.

### Correlation Analyses

**Figure 2:**
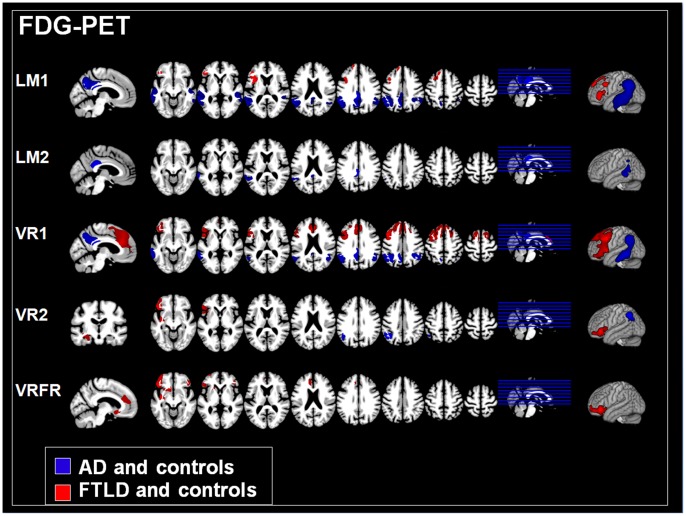
Memory performance and hypometabolism. Correlations between hypometabolism (FDG-PET) and each memory measure for AD patients and controls (blue) as well as FTLD patients and controls (red). LM: WMS *Logical Memory*, VR: WMS *Visual Reproduction*, 1: immediate recall, 2: delayed recall, FR: Forgetting rate. Results are only reported for those memory measures for which significant correlations in any region were found. Only clusters are shown that exceeded a significance threshold of p<0.001 uncorrected at voxel level and a cluster threshold of 2000 voxels. Left is left.

**Figure 3:**
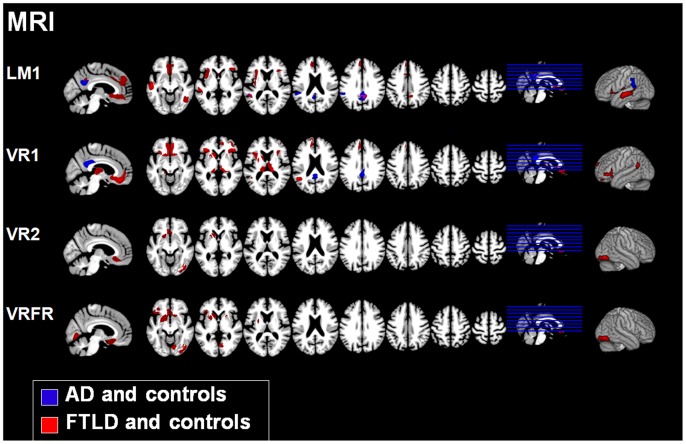
Memory performance and atrophy. Correlations between atrophy (MRI) and each memory measure for AD patients and controls (blue) as well as FTLD patients and controls (red). Areas of overlap are shown in violet (in LM1 only). LM: WMS *Logical Memory*, VR: WMS *Visual Reproduction*, 1: immediate recall, 2: delayed recall, FR: Forgetting rate. Results are only reported for those memory measures for which significant correlations in any region were found. Only clusters are shown that exceeded a significance threshold of p<0.001 uncorrected at voxel level and a cluster threshold of 2000 voxels. Left is left.

Figure 2 and Table 3 display the results of the correlation analyses between metabolism and memory performance in each dementia group and controls for each memory measure. Correlations between atrophy and memory performance are shown in Figure 3 and Table 4.

**Table 3:** Memory performance and hypometabolism.

Subtest	Regions	Cluster size	x	y	z	T-value
***AD and Controls***						
**LM1**	bilateral dorsal posterior cingulate gyrus,bilateral precuneus, bilateral retrosplenial cortex	13338	−5	−39	30	7.68
	left inferior parietal lobule, left middle and posterior superior/middle/inferior temporal gyrus	43495	−53	−52	36	6.14
	right posterior and middle superior temporal sulcus, right inferior, middleand superior temporal gyrus	8210	48	−35	2	5.01
	right inferior parietal lobule	8166	27	−49	42	4.34
**LM2**	bilateral dorsal posterior cingulate cortex	2477	−4	−38	26	5.46
	left posterior superior and middle temporal gyrus	4703	−68	−44	−2	4.32
**LMFR**		n. s.				
**VR1**	left inferior parietal lobule, left posterior middle and inferior temporal gyrus	23806	−34	−50	35	6.46
	left dorsal posterior cingulate cortex, left precuneus	7156	−16	−48	36	5.88
	right inferior parietal lobule, right temporo-parietal junction area	8970	37	−51	42	4.89
**VR2**	left inferior parietal lobule/angular and supramarginal gyrus	6038	−39	−55	38	4.97
**VRFR**		n. s.				
***FTLD and Controls***						
**LM1**	left posterior inferior frontal sulcus, left inferior frontal junction area	2384	−37	7	36	4.5
	left inferior frontal gyrus, left anterior superior insula	5097	−37	10	10	4.47
	left posterior superior frontal sulcus	2957	−14	46	39	4.14
**LM2**		n. s.				
**LMFR**		n. s.				
**VR1**	left inferior frontal gyrus (pars orbitalis, pars opercularis and parstriangularis), left posterior middle and superior frontal gyrus	45383	−45	13	41	6.66
	bilateral anterior midcingulate cortex, bilateral paracingulate cortex, bilateraldorsal frontomedian cortex, bilateral posterior superior frontal gyrus	10849	3	36	24	5.25
**VR2**	left inferior frontal gyrus (pars orbitalis and triangularis), left superioranterior insula	7049	−49	32	−6	6.18
	left anterior hippocampus, left inferior insula	3017	−30	−11	−14	5.56
**VRFR**	left inferior frontal gyrus (pars orbitalis and pars triangularis); left anteriorinsula, left caudate head, left ventral striatum, left basal forebrain	10776	−47	32	−8	6.05
	left inferior insula, left anterior hippocampus	3594	−36	−8	−13	5.72
	right inferior frontal gyrus (pars orbitalis)	2186	43	35	−3	4.54
	left pregenual anterior cingulate cortex	2128	−12	33	23	4.37

Statistical results for the correlations between each memory measure and hypometabolism (FDG-PET) for AD patients and controls as well as FTLD patients and controls. Cluster size is reported in voxels, voxel size was 1×1×1 mm.

**Table 4:** Memory performance and atrophy.

Subtest	Regions	Cluster size	x	y	z	T-value
***AD and Controls***						
LM1	left supramarginal gyrus, left posterior superior temporal gyrus	2279	−52	−46	23	4.81
	bilateral dorsal posterior cingulate cortex, bilateral inferior precuneus	3894	1	−50	29	4.48
LM2		n. s.				
LMFR		n. s.				
VR1	bilateral dorsal posterior cingulate cortex, bilateral inferior precuneus	4028	−2	−49	24	4.92
VR2		n. s.				
VRFR		n. s.				
***FTLD and Controls***						
LM1	left superior temporal sulcus, left superior and middle temporal gyrus	6207	−50	−25	−2	6.43
	right inferior frontal gyrus (pars orbitalis and pars triangularis), rightsuperior anterior insula	2470	42	32	5	5.68
	bilateral inferior precuneus	2322	3	−52	36	4.93
	right posterior inferior temporal gyrus	2286	46	−61	−13	4.91
	bilateral subgenual anterior cingulate cortex, bilateral ventromedialfrontal cortex	4102	−1	21	−3	4.74
	left superior anterior insula	4625	−33	9	4	4.72
	left paracingulate gyrus, left dorsal anterior frontomedian cortex	3241	−5	42	37	4.12
LM2		n. s.				
LMFR		n. s.				
VR1	right inferior frontal gyrus (pars orbitalis and pars triangularis), rightanterior superior insula	5386	41	35	−2	5.94
	bilateral thalamus, left posterior parahippocampal gyrus	7496	18	−27	7	5.68
	bilateral subcallosal area, bilateral ventromedial frontal cortex, bilateralcaudate head, left dorsal putamen, right anterior putamen, right ventralstriatum, right basal forebrain, left anterior insula, left inferior frontalcortex (pars orbitalis and pars triangularis)	19147	−37	35	−3	5.59
	left temporo-parietal junction area	2026	−47	−55	22	5.4
	left anterior dorsal frontomedian cortex, left paracingulate cortex,left superior frontal gyrus	2965	−11	48	24	4.35
VR2	right inferior occipital gyrus	2935	38	−76	−10	6.84
	bilateral subcallosal area, bilateral subgenual anterior cingulate cortex, bilateralcaudate head, bilateral anterior putamen, bilateral ventral striatum,bilateral basal forebrain	5078	−3	26	−10	4.18
VRFR	right inferior occipital gyrus	3623	38	−76	−10	7.1
	right lingual gyrus	2402	7	−70	−6	6.16
	left anterior insula, left inferior frontal gyrus (pars orbitalis and triangularis),left anterior putamen, bilateral ventral striatum, bilateral basal forebrain,bilateral rectal gyrus, bilateral subgenual anterior cingulate cortex,bilateral caudate head	18253	−42	24	−4	5.2

Statistical results for the correlations between each memory measure and atrophy (MRI) for AD patients and controls as well as FTLD patients and controls. Cluster size is reported in voxels, voxel size was 1×1×1 mm.

#### FDG-PET: AD patients and controls

Performance in the immediate recall of verbal information (LM1) was correlated with glucose metabolism in the bilateral parieto-mesial cortex (precuneus, retrosplenial and cingulate cortex) and in the lateral parietal and temporal regions of both hemispheres, though much more extensive in the left. Delayed verbal recall (LM2) was also associated with bilateral parieto-mesial and left lateral temporal glucose metabolism. Forgetting rates (LMFR) did not correlate with glucose utilization in any region. Performance in immediate visual recall (VR1) was correlated with glucose metabolism in left parieto-mesial regions (cingulate gyrus, precuneus) and in temporo-parietal regions in both hemispheres, with a focus in the left hemisphere. Performance in the delayed recall of visual information (VR2) was correlated with glucose metabolism in the left inferior parietal lobe. We did not find any correlations between glucose metabolism and visual forgetting rates (VRFR).

#### FDG-PET: FTLD patients and controls

Performance in the immediate recall of verbal information (LM1) was correlated with glucose metabolism especially in the left inferior frontal cortex and in the left anterior insula. Neither delayed verbal recall (LM2) nor verbal forgetting rates (LMFR) were correlated with glucose metabolism in any region. Correlations between the immediate recall of visual information (VR1) and glucose metabolism were also accentuated in the frontal cortex, though much more extensive compared to the verbal domain (LM1). In addition to the left superior, middle and inferior frontal cortices, correlations were also found in the frontomedian cortex including the anterior cingulate gyrus. With respect to delayed visual recall (VR2), we found that glucose metabolism and test performance were again positively correlated in the left inferior frontal cortex and in the insula, and additionally in the left anterior hippocampus. Forgetting rates of visual information were negatively correlated with glucose utilization in the lateral inferior frontal cortices bilaterally and in the left insula, anterior cingulate cortex, basal ganglia (esp. caudate), anterior hippocampus and basal forebrain.

#### MRI: AD patients and controls

Correlations between performance in the immediate recall of verbal information (LM1) and GM volume were found in bilateral parieto-mesial areas (precuneus, cingulate gyrus) as well as in the left inferior parietal and superior temporal cortex. We did not find any correlations between the scores in the delayed verbal recall (LM2) or verbal forgetting rates (LMFR), respectively, and gray matter volumes in any region. Similarly, lower performance in the immediate visual recall (VR1) was associated with lower GM volume in parieto-mesial regions (precuneus, posterior cingulate). Again, we did not find any correlations between the scores in delayed verbal recall (LM2) or verbal forgetting rates (LMFR), respectively, with GM volumes in any region.

#### MRI: FTLD patients and controls

Deficits in immediate verbal recall (LM1) were correlated with atrophy in the precuneus bilaterally, the right inferior frontal cortex, the left insula and the left dorsal frontomedian cortex. Further correlations were found in the bilateral orbitofrontal cortices as well as the anterior cingulate cortex. Performance in the immediate recall of visual information (VR1) was correlated with GM volume in the inferior frontal and the orbitofrontal cortices bilaterally and in the left parahippocampal gyrus as well as in the basal ganglia (caudate, putamen), thalamus and subcallosal area bilaterally. Correlations between performance in the delayed recall of visual information (VR2) and brain volume were mainly found in bilateral frontal cortical (anterior cingulate) and subcortical areas (basal ganglia, subcallosal area), and in the right inferior occipital gyrus. Visual forgetting rates (VRFR) were negatively associated with GM volume in a large cluster comprising the left inferior frontal gyrus, the left insula, the anterior cingulate cortices, basal forebrain and basal ganglia bilaterally as well as in an area in the right occipital cortex.

#### Conjunction analysis

We found an overlap of atrophy (MRI) with respect to the LM1 subtest between AD and control subjects, and FTLD and control subjects, respectively, in the bilateral posterior cingulate cortex and the left superior temporal gyrus (851 voxels, corresponding to 13.8% of all voxels in AD and controls and 3.4% of all voxels in FTLD and controls, respectively, see overlapping regions in Figure 3/LM1). No further overlaps were observed in any of the remaining analyses.

## Discussion

In the present study, we investigated the neural correlates of immediate and delayed verbal and visual memory in patients with early AD and FTLD with respect to both PET hypometabolism and MR brain atrophy. To our knowledge, this is the first study that investigates both patient groups with respect to different memory domains (verbal, visual, forgetting rates) and different imaging markers (glucose metabolism, GM volume) in a whole-brain approach.

### Lesion Patterns in AD and FTLD

Group comparisons in FDG-PET and MRI between each of the dementia groups and control subjects largely replicate previous findings from the literature. Hippocampal atrophy in the AD group compared to controls in connection with GM loss in medial and lateral parietal regions is a common finding [34], [35]. The absence of hypometabolism in the hippocampus seems more surprising as there are diverging findings in the literature on this issue [64]. Regional hypometabolism might have disappeared in our study, because we corrected FDG-PET data for partial volume effects according to atrophy – a procedure that is uncommonly applied in the literature. Whereas the finding of atrophy in fronto-median and fronto-lateral cortices and in the basal ganglia in FTLD is expected with respect to previous studies [20], [49], [50], [60], [65], [66], hippocampal atrophy seems puzzling at first sight. However, there are a number of studies which report volume reductions in the hippocampus in FTLD [18]–[22], [50], [51], also in pathologically validated FTD [16]. In a very recent study, hippocampal atrophy did not differ between AD and FTD patients which lead the authors to the general conclusion that the extent of hippocampal atrophy is not an efficient diagnostic marker in order to distinguish AD from FTD [66]. This latter study further confirms the view that memory may be more severely affected than previously assumed in these patients.

### Memory Performance in AD and FTLD

With respect to memory test performance, the two dementia subgroups did not differ from each other, although both performed significantly lower than controls in all test measures, except for visual forgetting rates where FTLD patients did not differ from controls. This latter finding may point to slight differences between the groups, probably due to stronger visuospatial problems interfering with visual memory in the AD, but not the FTLD group [14], [18].

Findings of considerable memory impairments in early stages of FTLD have already been reported which raised the questioned whether FTLD and AD patients can be clearly distinguished on the basis of memory test performance (see Introduction). The results of our study support these findings as both dementia groups did not differ in their abilities to retrieve information that has been encoded initially, since even forgetting rates did not differ between the groups. However, our results do not allow deciding whether the low forgetting rates in AD and FTLD are caused by the same mechanisms or whether both groups might nevertheless differ in their abilities to consolidate information as our study did not include recognition trials [11], [12], [24].

### Neural Correlates of Memory in AD and FTLD

Aside from small atrophy overlaps in the LM1 subtest (immediate recall of verbal information) as revealed by a conjunction analysis, we found that similar decreases in memory performance between the two dementia groups had completely different underlying neural substrates: The parieto-mesial cortex (precuneus, retrosplenial and posterior cingulate cortex) correlated with memory performance (both verbal and visual) only when the AD group was included. This finding corroborates studies which highlight the role of these areas in the development of AD (see Introduction) as well as in the context of memory functions, such as the retrieval of (episodic) memories in both healthy control subjects (see refs [67]–[68] for reviews) and in patients with memory deficits such as AD patients [48] or patients with transient global ischemia [69]. One anatomical explanation for this role might be the high interconnectivity between the medial parietal lobe and medial temporal structures [68], [70]. In contrast, when the FTLD group was included, memory performance was primarily associated with metabolism and GM volume in frontal cortical (esp. lateral and medial frontal cortex) and subcortical structures (insula, basal ganglia, anterior cingulate, and basal forebrain). These results are in line with earlier findings that these regions are primarily affected in FTLD, a result which was also confirmed in our group comparisons. Especially the inferior frontal cortex has been shown to play an important role in encoding and retrieving information [71]–[73]. Our results suggest that further areas are involved in FTLD which are crucial for memory functions such as the anterior cingulate cortex [71], the basal ganglia [74], and the basal forebrain [75], [76] which can all lead to severe amnesia in the case of damage. Thus, although lesion patterns differed between AD and FTLD, structures crucial for memory functions seemed to be affected in both diseases.

### Limitations of the Study

Finally, there are some limitations of our study. First of all, our diagnostic classification was purely based on clinical criteria. Further studies are needed to dissociate memory networks in FTLD subtypes including histopathological validation.

Secondly, the small sample sizes (especially in the FTLD group), principally limit a generalization of the results to the different disease populations. Furthermore, the small sample sizes do not allow dissociating possible differences in the memory related networks between the FTLD subgroups (FTD, SD, mixed type). Such differences as well as differences in memory performance might be expected according to the literature (see Introduction). Assigning all these patients to one group (which was not possible otherwise due to the small number of subjects) might have lead to the high variability thereby obscuring possible differences between the FTLD and the AD group.

A reason why we did not observe differences between AD and FTLD patients in any of the neuropsychological tests might be a lack of power in our study resulting from the small sample sizes. In fact, estimations of effect sizes for the differences in neuropsychological test performance for both groups of patients indicated small to medium sized effects for all of the neuropsychological tests (between r = .06 to r = .36).

Another potential source of limitation for the interpretation of differential spatial networks showing a correlation with neuropsychological performance might be a lack of power in one of the conditions. However, we do not think that the observed distinction in networks can be explained by a lack of power, for the following reasons: First of all, a lack of power in one of the conditions (either correlations of imaging data with memory scores only in AD and controls or only in FTLD and controls) would rather result in the observation of a specific network in one but not in the other condition. In contrast, what we found were two distinct networks showing similarly significant correlations with the neuropsychological performance in both analyses. Secondly, we found even slightly higher t-values in some of the correlation analyses with the smaller number of subjects (FTLD and controls) which is the opposite of what one would expect if the difference in results would be attributable to a lack of power.

In addition, we cannot completely rule out the objection that subjects with cognitive complaints were not ideal controls. Including these subjects as controls might have underestimated the deficits in the two dementia groups. Nevertheless, we think that it was justified to include them as a comparison group for the following reasons: In the examination, it became clear that these subjects experienced an age-related -though not abnormal according to our assessment- cognitive decline which concerned them (e. g. that they were not as fast at work anymore or could not memorize as many details compared to earlier ages). None of them had a CDR score greater than 0.5 which is also found frequently in random samples from the normal population. An earlier study e. g. reported that 23% of the subjects in a random sample had a CDR of 0.5 [77], although the mean age in these subjects was higher than in ours. Certainly, one general limitation of studies such as ours is that, for ethical reasons, invasive techniques such as PET should not be applied without clinical justification.

Furthermore, we do not claim that early AD and FTLD patients have a completely identical profile with respect to their memory abilities. Our results only show that widely used neuropsychological test measures of immediate and delayed recall may not differentiate between the groups, even if additional parameters such as forgetting rates are applied. We cannot exclude that other test parameters (such as a recognition paradigm), experimental procedures (e. g. on source memory) or more naturalistic tasks (such as incidental encoding) may reveal differences (see ref [27] for a review).

Last but not least: We have used the term “network” very generally. In fact, we have only identified patterns of brain areas in which the extent of damage is correlated with a task performance. However, we have used a rather “static” methodological approach (atrophy and metabolism at rest) which did not allow disentangling the dynamic interplay of these areas while a specific function is carried out in a lesioned brain. This would be an aim for further studies using methods such as DTI, task-related fMRI or resting state connectivity [78]–[80].

### Conclusion

We have shown that memory test performance may not differentiate between early AD and FTLD, but that the underlying memory networks can nevertheless be clearly dissociated with respect to both hypometabolism and atrophy. Furthermore, this dissociation does not depend on a priori hypotheses with respect to specific brain areas, but can also be shown in a data-driven, whole brain approach. This finding contributes to a more profound understanding of how memory processes are connected to the degeneration of the underlying neural networks.

With respect to clinical practice, even severe memory problems should not automatically exclude the diagnosis of FTLD as this might lead to misclassifications [26]. With respect to the classification of clinical dementia syndromes, our data suggest that respective algorithms should include a diversity of clinical as well as imaging parameters in order to reveal the crucial differences.

More generally, our data speak in favour of a view according to which functions are not strictly “localized” in the brain but are carried out by distributed networks. This view leads to interesting predictions for how the brain deals with lesions [79]. Neurodegenerative dementias are “paradigmatic” for this network view as these diseases crucially involve lesions of central nexuses (“hubs”) [80]. This leads to a loss of connectivity to a degree which cannot be compensated anymore by the rest of the brain and therefore leads to the far-reaching disabilities in dementing illnesses.
